# The Impact of a Vegan Diet on Many Aspects of Health: The Overlooked Side of Veganism

**DOI:** 10.7759/cureus.35148

**Published:** 2023-02-18

**Authors:** Atul Bali, Roopa Naik

**Affiliations:** 1 Internal Medicine / Nephrology, Geisinger Health System, Wilkes-Barre, USA; 2 Medicine, Geisinger Commonwealth School of Medicine, Scranton, USA; 3 Medicine, Geisinger Medical Center, Danville, USA; 4 Internal Medicine, Geisinger Health System, Wilkes-Barre, USA

**Keywords:** bone mineral density, children adolescents, food and nutrition, maternal and fetal outcome, mental wellbeing, vegan diet

## Abstract

Vegetarianism in any of its various forms, particularly veganism, has been increasing in popularity over the past few years, especially among the young population in the United States. While several studies have shown that a vegan diet (VD) decreases the risk of cardiometabolic diseases, such as cardiovascular disease, type 2 diabetes mellitus, obesity, and non-alcoholic fatty liver disease, veganism has been associated with adverse health outcomes, namely, nervous, skeletal, and immune system impairments, hematological disorders, as well as mental health problems due to the potential for micro and macronutrient deficits. The goal of this review article is to discuss the current literature on the impact and long-term consequences of veganism on vulnerable populations, including children, adolescents, pregnant and breastfeeding women, and fetal outcomes in strict vegan mothers. It also focuses on the many deficiencies of the vegan diet, especially vitamin B12, and the related increased risk of malignancies.

## Introduction and background

Vegetarianism in various forms has gained widespread popularity in recent years. These types include vegans, who adhere to the most stringent dietary restrictions, omitting all animal-source foods and their by-products from the diet. Others include lactovegetarians (no meat, fish, or eggs but do consume dairy goods), ovo-vegetarians (no meat, fish, or dairy products but do consume eggs), lacto-ovo-vegetarians (no meat but do consume eggs and dairy products), and pescatarians (no meat except fish and shellfish) [1,2]. There has been growing interest in dietary habits given the worsening obesity epidemic and obesity-related health concerns [3,4]. Obesity is an established risk factor for diabetes mellitus, which, in turn, is an independent risk factor for coronary artery disease [5]. While studies have shown that a vegan diet (VD) may be associated with improved health outcomes [6,7], the negative health repercussions of these food preferences, on the other hand, are rarely highlighted, and veganism may be associated with negative health effects due to nutritional deficiencies.

**Figure 1:**
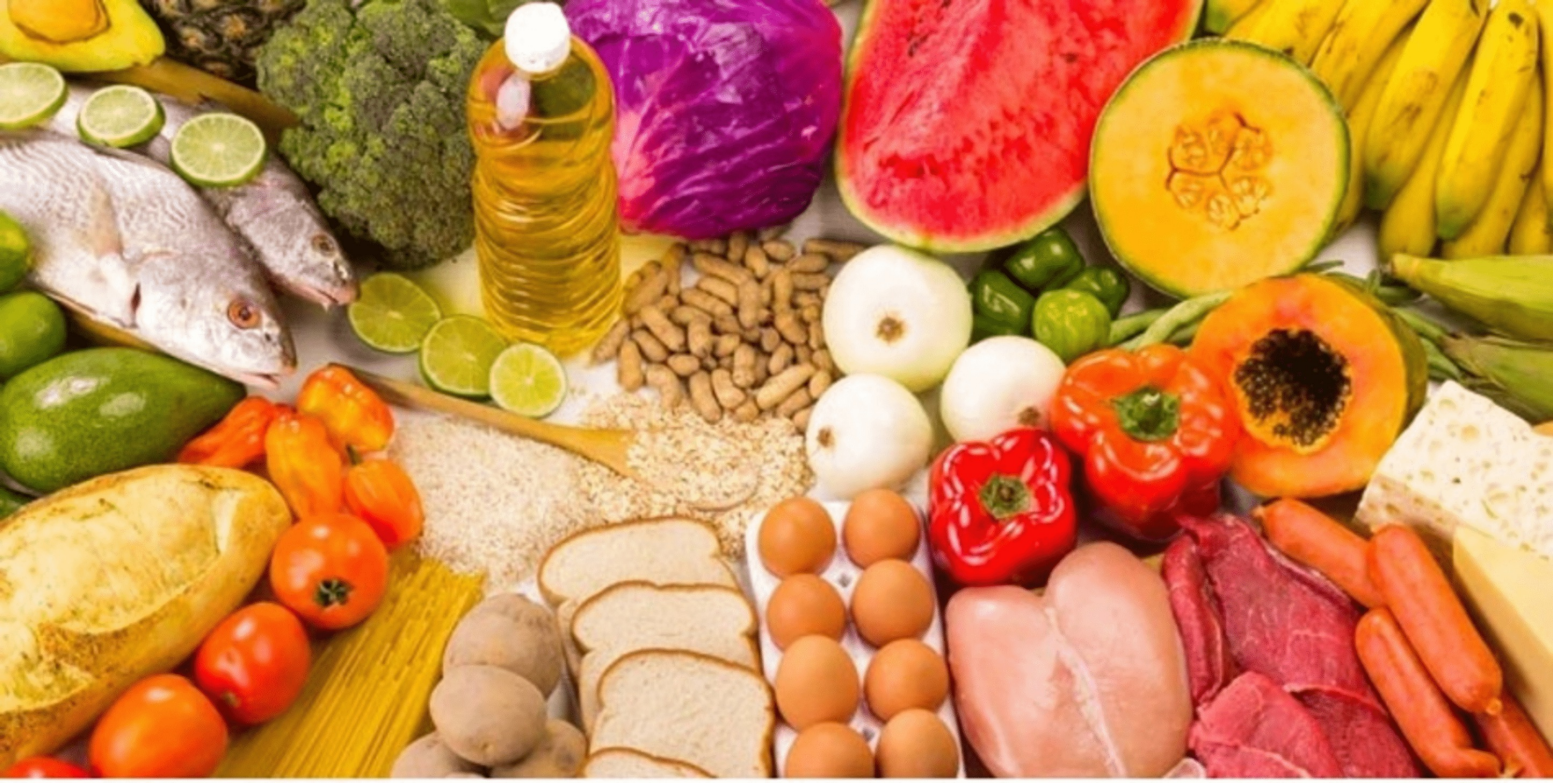
Image of diverse dietary elements constituting a balanced diet with proportion sizes

Additionally, vegans have a greater prevalence of mental health problems, which may lead to a poorer quality of life. An optimal diet should be balanced, consisting of lean meat, nuts, fresh fruits and vegetables, and olive oil (Figure 1) [8,9]. A wholesome diet is essential in maintaining a healthy gut flora, which in turn is pivotal in avoiding inflammatory disorders [10-13]. The primary aim of this review will be to draw attention to the current literature associated with veganism, including the side effects of practicing a VD and long-term consequences for a variety of populations, including adults, adolescents, pregnant and lactating women, and the fetal outcomes of vegan mothers.‬‬‬‬‬‬

## Review

Protein

A recent systematic study examined the intake and adequacy of the VD in terms of macro and micronutrient intake in the adult European population. The study found that vegans consumed the least total protein compared to other diet groups, confirming concerns that VD may include insufficient protein, particularly in instances where legume, seed, and nut consumption is limited [14]. Vegans consume fewer essential amino acids than non-vegans [15]. Plant proteins are less digestible (50-70%) than animal proteins, and food processing methods like heating may further reduce digestibility. According to the WHO, animal proteins are considered complete proteins and have higher biological value, protein efficiency ratio, net protein utilization, and, ultimately, have a higher Protein Digestibility Corrected Amino Acid Score (PDCAAS) compared to plant proteins [16,17]. By and large, soy proteins constitute a significant protein source for most vegans [18].

Vitamin B12

Low vitamin B12 intake is a significant problem in vegan diets due to the exclusion of vitamin B12-rich foods such as meat, poultry, and eggs. A lack of vitamin B12 has been linked to neurologic and hematologic problems [19]. Low vitamin B12 intake has serious clinical consequences, although deficient symptoms appear gradually over time. High folate levels may also partly and temporarily obscure some of the typical vitamin B12 hematological manifestations. To prevent vitamin B12 deficiency, vegans must get their levels checked regularly and meet their daily requirements via supplements or fortified foods.

A growing body of research indicates that inadequate B12 consumption may contribute to carcinogenesis. Vitamin B12 deficiency increases uracil misincorporation, impairing DNA synthesis and genomic instability. Global hypomethylation of DNA is a characteristic of early carcinogenesis. Thus, if not adequately replaced, VD may inadvertently lead to cancers [20]. Wu et al found that blood B12 levels were substantially lower in menopausal and postmenopausal breast cancer patients, and patients with the lowest B12 levels had an elevated risk of breast cancer [21]. Reduced B12 levels have also been linked to an increased risk of cervical and gastrointestinal tract malignancies [22-24]. As a result, B12 supplementation is imperative for vegans due to the extensive and irreversible detrimental effects of the deficiencies.

Other minerals and micronutrients

Vegans have greater iron needs than other diet patterns [25], mainly because non-heme iron from plant-based foods is less bioavailable, as absorption is hindered by whole grains, legumes, and nuts due to their phytic acid content [26]. Vegans also have a zinc deficit. While meat, dairy, and eggs contain zinc, some zinc-rich plant foods (e.g., nuts, seeds, and whole grains) have poor bioavailability owing to the presence of phytate, which inhibits absorption in the gut [16]. Inadequate zinc consumption may be associated with mental health problems (e.g., depression), dermatitis, diarrhea, and alopecia, all of which are more prevalent among vegans [27,28]. Selenium insufficiency has also been seen among vegetarians.

Vitamin D, calcium, bone mineral density, and risk of fractures

Numerous studies have shown that vegans consume insufficient calcium and vitamin D, not only owing to the absence of dairy products but also due to calcium bioavailability problems in plant-based diets [28]. Vitamin D insufficiency exacerbates calcium shortage further owing to impaired intestinal absorption. After adjusting for socioeconomic variables, lifestyle covariates, and body mass index (BMI), a recent study reported that as compared to meat-eaters, there was an increased risk of hip fractures observed in vegetarians (HR 1.25; CI 1.04-1.50), vegans (2.31; 1.66-3.22), and fish eaters (1.26; 1.02-1.54) [29]. Vegans also had a greater incidence of overall fracture (1.43; 1.20-1.70), leg fractures (2.05; 1.23-3.41), and fractures in other major sites (1.59; 1.02-2.50). The higher risk of fractures may be related to vegans' significantly lower calcium intake, reduced dietary protein intake, and lower BMI [30-32].

Mental health

The most recent systematic review [33], which included eighteen studies, compared meat abstainers versus meat eaters in terms of mental health. The research included 160,257 individuals (85,843 females and 73,232 men) from various geographic areas, including 149,559 meat eaters and 8584 meat abstainers (aged 11 to 96 years). Eleven of the 18 studies found that meat-free diets were linked with worse psychological health, four were inconclusive, and three found that meat-free diets resulted in improved results. The most thorough research found that meat-avoiders (i.e., "full vegetarians") had a 7.4%, 24.1 %, and 35.2% 1-month, 12-month, and lifetime prevalence of unipolar depressive disorders, respectively. In contrast, meat consumers had a much lower prevalence: 6.3%, 11.9%, and 19.1%. Similarly, the 1-month, 12-month, and lifetime prevalence of anxiety disorders for meat abstainers were much higher at 20.4%, 31.5%, 31.5%, and 10.7%, 17.0%, and 18.4% in the meat eaters respectively. The study highlights the high incidence of mental health problems among vegans, emphasizing the vital need of increasing awareness of these illnesses to facilitate early intervention. Women notably appeared to be adversely impacted by mental disorders such as stress [34-36].

Orthorexia

Orthorexia nervosa (ON) is defined as a fixation on health-conscious eating behavior [37]. It involves obsessive (compulsive) food decisions, self-imposed anxiety, self-punishment, and increasingly extreme limitations. As a result of diet-related concerns, individuals develop dietary restrictions, lack of food pleasure, inflexible and rigid eating behaviors, and ritual activities surrounding food preparations. Vegetarian, and vegan, women are more prone than males to have disordered eating attitudes and practices [38].

Mortality

Although some studies indicate a reduction in mortality associated with vegetarianism and VD, the larger body of evidence indicates that the health benefits associated with vegetarianism may be due to other “lifestyle” factors associated with socioeconomic statuses, such as adequate physical activity, low alcohol, and drug consumption, or avoidance of tobacco products. Recently, Johnston et al. argued that the evidence supporting public health recommendations to reduce or eliminate meat intake was based on questionable studies and "inappropriate analysis" [39]. This argument and the growing body of contrasting and conflicting findings create a conundrum for doctors and policymakers alike.

Effects on children and adolescents

Adolescents are also known to show a strong preference for VD. A balanced diet is critical for children and adolescents to meet their bodies' rising demands during the growth spurt, rendering the implications of VD in this vulnerable age more intriguing. A recent study examined the anthropometry, dietary intakes, and nutritional status of 149 vegetarians, 115 vegans, and 137 omnivore children and adolescents using a cross-sectional design (6-18 years old, mean age: 12.7 ± 3.9 years) [40]. Vegetarians and vegans consumed more carbohydrates than omnivores (p = 0.0002). Vegetarians (p = 0.02) had the lowest protein consumption, however, vitamin B2, D3, HDL-C, and triglyceride blood concentrations did not differ between diet groups. The authors concluded that there are no specific nutrient concerns among vegetarian, vegan children and adolescents compared to omnivores. The study's cross-sectional design and lack of representativity should be considered when interpreting the findings.

In contrast to the above study, subsequent cross-sectional studies showed that vegetarian and/or vegan children had a lower bone mineral density (BMD) [41,42]. Desmond et al. observed that vegetarians and vegans were shorter than omnivores (-0.32 and -0.57 height z scores, respectively), but the difference was non-significant in vegetarians [42]. The research showed that after controlling for body size, vegan children had substantially lower vitamin D levels and BMD than omnivores. It is suggested to maximize childhood BMD to promote peak BMD and therefore reduce the risk of osteoporosis and fracture in adulthood. The authors concluded that vegans had lower BMDs even when body and bone size were taken into consideration. It does not seem to be ideal to start puberty, a period when bone-specific nutrition requirements are greater, with an already established BMD deficiency. If such deficiencies continue throughout adolescence as a result of a diet, they may raise the likelihood of poor bone outcomes later in life. Prospective longitudinal studies are required to better understand the consequences of VD on children and adolescents.

Effects on pregnancy, fetal outcomes, and lactation

Optimal fetal growth requires balanced maternal nutrition during pregnancy. Mothers on rigorous VD are at risk of vitamin insufficiency, which can lead to poor fetal outcomes. A recent study included 273 women, including 112 omnivores, 37 fish eaters, 64 lacto-ovo-vegetarians, and 60 vegans, respectively [43]. In comparison to an omnivorous diet, the vegan diet was substantially linked with an elevated risk of small-for-gestational-age infants (RR = 5.9, 95 percent CI, 1.2-21.8). All the groups had a similar incidence of preterm births. Birthweight in vegans was lower compared to lacto-ovo-vegetarians (3015 ± 420 g vs. 3285 ± 482 g, P = 0.004) and to omnivores (3328 ± 495 g, P < 0.001) but not to fish-eaters. Vegans also had a lower mean gestational weight gain compared only to omnivores (11.6 ± 4.2 kg vs. 14.3 ± 4.6 kg, P = 0.001). A review of 13 low and middle-income nations found low docosahexaenoic acid levels in breast milk in mothers on plant-based diets but greater in the fish-eating population [44].

Maternal B12 status influences their offspring’s B12 levels and is an independent risk factor for neural tube defects (NTD) [45]. Studies have shown an association between low B12, low birth weight, and pre-term delivery [46]. A Chinese study associated increased maternal pickled vegetable consumption with NTD due to excessive nitrate, nitrite, and N-nitroso compound content [47]. They found that eating pickled vegetables more frequently (>6 meals/week) increased the risk of NTD. The investigators also found that maternal consumption of meat, eggs, or milk (>1 meal/week) reduced the risk of NTD. Vegan mothers may have poor prenatal nutritional status, resulting in low maternal fat reserves for breastfeeding. The postpartum nutritional profile of vegetarian mothers declines without sufficient energy intake, thus maternal nutritional reserves are lost to promote infant normal development. 

Providers should evaluate a woman's nutrition and energy consumption frequently. Women on restrictive diets may need to take supplements or eat fortified foods to meet the required needs throughout pregnancy and breastfeeding. Plant-based diets during pregnancy and breastfeeding need a heightened awareness of the importance of consuming all necessary nutrients and vitamin supplements, as recommended by international guidelines [48].

## Conclusions

While veganism has been shown to decrease the risk of cardiovascular and metabolic syndrome, it also carries the potential for micro- and macronutrient deficits. It should be noted that vegans often have better socioeconomic levels, live a healthier lifestyle with more physical exercise, and tend to smoke less compared to non-vegetarians, making it difficult to isolate the effects of veganism in observational research. Existing research is often skewed by selection bias, which is when the study sample is chosen based on prior eating patterns and such studies are often recruited in environments with a high level of health awareness. Our review focuses on the impact of veganism on vulnerable populations, including children, adolescents, pregnant and breastfeeding women, and fetal outcomes in strict vegan mothers. Vegans should be closely monitored and treated for nutritional deficiencies, in order to mitigate any long-term negative health outcomes. Given the growing interest in diets without animal protein intake in the general population, it is crucial, now more than ever, to have a clear understanding of both the risks and benefits of such diets among clinicians, policymakers, and the public.
