# Correction: The Impact of a Vegan Diet on Many Aspects of Health: The Overlooked Side of Veganism

**DOI:** 10.7759/cureus.c216

**Published:** 2025-04-10

**Authors:** Atul Bali, Roopa Naik

**Affiliations:** 1 Internal Medicine / Nephrology, Geisinger Health System, Wilkes-Barre, USA; 2 Medicine, Geisinger Commonwealth School of Medicine, Scranton, USA; 3 Medicine, Geisinger Medical Center, Danville, USA; 4 Internal Medicine, Geisinger Health System, Wilkes-Barre, USA

This article has been corrected by the Editors-in-Chief to replace references 34-36 as the articles that were originally references were inadvertently included. The journal regrets that this error was not identified and corrected prior to publication.

